# High Temperatures Enhanced Acute Mortality Effects of Ambient Particle Pollution in the “Oven” City of Wuhan, China

**DOI:** 10.1289/ehp.10847

**Published:** 2008-07-09

**Authors:** Zhengmin Qian, Qingci He, Hung-Mo Lin, Lingli Kong, Christy M. Bentley, Wenshan Liu, Dunjin Zhou

**Affiliations:** 1 Department of Public Health Sciences, Pennsylvania State University College of Medicine, Hershey, Pennsylvania, USA; 2 Geisinger Center for Health Research, Danville, Pennsylvania, USA; 3 Wuhan Academy of Environmental Science, Wuhan, China; 4 Mount Sinai School of Medicine, New York, NY, USA; 5 Wuhan Environmental Monitoring Center, Wuhan, China; 6 Wuhan Centres for Disease Prevention and Control, Wuhan, China

**Keywords:** air pollution, China, health effect, mortality, temperature

## Abstract

**Background:**

We investigated whether the effect of air pollution on daily mortality is enhanced by high temperatures in Wuhan, China, using data from 2001 to 2004. Wuhan has been called an “oven” city because of its hot summers. Approximately 4.5 million permanent residents live in the 201-km^2^ core area of the city.

**Method:**

We used a generalized additive model to analyze pollution, mortality, and covariate data. The estimates of the interaction between high temperature and air pollution were obtained from the main effects and pollutant–temperature interaction models.

**Results:**

We observed effects of consistently and statistically significant interactions between particulate matter ≤ 10 μm (PM_10_) and temperature on daily nonaccidental (*p* = 0.014), cardiovascular (*p* = 0.007), and cardiopulmonary (*p* = 0.014) mortality. The PM_10_ effects were strongest on extremely high-temperature days (daily average temperature, 33.1°C), less strong on extremely low-temperature days (2.2°C), and weakest on normal-temperature days (18.0°C). The estimates of the mean percentage of change in daily mortality per 10-μg/m^3^ increase in PM_10_ concentrations at the average of lags 0 and 1 day during hot temperature were 2.20% (95% confidence interval), 0.74–3.68) for nonaccidental, 3.28% (1.24–5.37) for cardiovascular, 2.35% (−0.03 to 4.78) for stroke, 3.31% (−0.22 to 6.97) for cardiac, 1.15% (−3.54% to 6.07) for respiratory, and 3.02% (1.03–5.04) for cardiopulmonary mortality.

**Conclusions:**

We found synergistic effects of PM_10_ and high temperatures on daily nonaccidental, cardiovascular, and cardiopulmonary mortality in Wuhan.

Extreme temperatures are associated with increased daily mortality in many regions of the world (Patz and Khaliq 2002). Because human activity is likely to increase overall global average temperatures, research efforts have focused on the health effects of exposure to high temperatures and heat waves in summer. In the United States, increased mortality during high-temperature days has been extensively investigated. Semenza et al. (1996) reported that a heat wave in Chicago, Illinois, in 1995 was associated with an increase in the death rate among socially isolated people who had no air conditioning. In studies of multiple U.S. cities, similar results were reported (Curriero et al. 2002). In Europe, excess mortality during high-temperature days has also been noted. Le Tertre et al. (2006) also reported an association between the 2003 heat wave in France and increases in all causes of mortality in nine French cities. Stafoggia et al. (2006) explored vulnerability to heat-related mortality in four Italian cities: Bologna, Milan, Rome, and Turin. The populations particularly vulnerable to high summer temperatures were the elderly, women, widows and widowers, those with particular medical conditions, and those in nursing homes and health care facilities.

Air pollution is also associated with increased daily mortality (Pope 2000). A large number of daily mortality time-series analyses have provided sufficiently convincing evidence that nonaccidental mortality, including cardio-pulmonary mortality, is associated with ambient particulate matter (PM) exposure in the United States (Ostro et al. 2007), Canada (Burnett et al. 2000), Rome (Forastiere et al. 2007), China (Kan et al. 2007), Korea (Lee et al. 2000), Greece (Katsouyanni et al. 1997), and Chile (Cakmak et al. 2007). The estimated effect is generally in the range of 1.0–8.0% excess deaths per 50-μg/m^3^ increments in 24-hr average concentrations of particulate matter ≤ 10 μm in aerodynamic diameter (PM_10_) (Schwartz and Zanobetti 2000).

Although the independent impacts of high temperature and air pollution on daily mortality have been widely explored, few studies have examined the interaction between high temperature and air pollution (Samet et al. 1998). Investigating the effects of the synergy between air pollution and high temperature on mortality, although desirable, is difficult, because a suitable study site is not easily available. The Chinese city of Wuhan, however, provides an opportunity to examine these synergistic effects; it has been called an “oven” city because of its extremely hot summers. Previous studies in Wuhan (He et al. 1993; Qian et al. 2004) have shown high air pollution levels, with concentration ranges wider than those reported in the published literature for other locations. Therefore, we tested the hypothesis that temperature extremes modify the mortality effects of air pollution.

## Methods

### Study area and population

Wuhan is the capital of Hubei Province, which is located in the middle of the Yangzi River delta, at 29°58′ −31°22′ north latitude and 113°41′ −115°05′ east longitude. Its population is approximately 7.5 million people, of whom approximately 4.5 million reside in nine urban core districts within an area of 201 km^2^. Wuhan has a subtropical, humid, monsoon climate with a distinct pattern of four seasons. Its average daily temperature in July is 37.2°C, and the maximum daily temperature often exceeds 40°C. The major industries in Wuhan include ferrous smelters, chemical plants, power plants, and machinery plants. The major sources of air pollution in the city are motor vehicles and the burning of coal for domestic cooking, heating, and industrial processes.

### Data sources

Mortality data from 1 July 2000 to 30 June 2004 were obtained from the Wuhan Centres for Disease Prevention and Control (WCDC). The government requires that a decedent’s family obtain a death certificate from a hospital or a local community clinic to remove the deceased person from the government-controlled household registration. The local WCDC issues two copies of the death certificate according to the certificate from the hospital or the clinic. One copy is submitted to the public safety department to stop the decedent’s address registration, and the other copy is used for the cremation.

The WCDC electronically archives all death certificates. In 1992, the WCDC became the first center in China to standardize its system for mortality data collection. The system’s requirements are as follows: *a*) mortality data must be validated four times per year; *b*) death events collected from the WCDC must conform with those collected from the Wuhan Police Department; *c*) no data may be missing from any death certificate; *d*) unclear causes and diagnosis may not constitute > 2% of deaths in urban districts; and *e*) a correct coding rate of > 98% must be achieved for cause-specific deaths. For deaths that occurred before 1 January 2003, the *International Classification of Disease, Ninth Revision* [ICD-9; World Health Organization (WHO) 1978] codes were applied; for deaths that occurred after 31 December 2002, ICD-10 (WHO 1993) codes were applied. Total mortality was divided into the following major causes: non-accidental mortality (ICD-9 codes 1–799; ICD-10 codes A00–R99), cardiovascular diseases (ICD-9 codes 390–459; ICD-10 codes I00–I99), stroke (ICD-9 codes 430–438; ICD-10 codes I60–I69), cardiac diseases (ICD-9 codes 390–398 and 410–429; ICD-10 codes I00–I09 and I20–I52), respiratory diseases (ICD-9 codes 460–519; ICD-10 codes J00–J98), and cardiopulmonary diseases (ICD-9 codes 390–459 and 460–519; ICD-10 codes I00–I99 and ICD-10 J00–J98). The Human Subject Protection Office of the Penn State College of Medicine approved the current study protocol.

Pollution data were collected by the Wuhan Environmental Monitoring Center (WEMC) and certified by the U.S. Environmental Protection Agency. Daily concentrations of PM_10_, sulfur dioxide, nitrogen dioxide, and ozone (8-hr mean concentrations, 1000–1800 hours) were collected for the study period. The monitoring system strictly followed the quality assurance/quality control procedure set by the State Environmental Protection Administration of China (1992). Briefly, the WEMC conducts regularly scheduled performance audits and precision checks on the air-monitoring equipment. Quarterly performance audits are also conducted to assess data accuracy. PM_10_ measurements were collected using PM_10_ beta attenuation mass monitors, (model 7001); SO_2_ measurements were collected using an ultraviolet fluorescence SO_2_ analyzer (model 4108); NO_2_ measurements were collected using a chemiluminescent NO_2_ analyzer (model 2108); and O_3_ measurements were collected using an ultraviolet photometry O_3_ analyzer (model 1008), all from Dasibi Environmental Corporation (Glendale, CA, USA). Meteorologic data were provided by the Wuhan Meteorological Administration.

### Statistical methods

We used quasi-likelihood estimation within the context of the generalized additive models (GAMs) to model the natural logarithm of the expected daily death counts as a function of the predictor variables (Hastie and Tibshirani 1990). We examined the effect estimates for each pollutant at 0-, 1-, 2-, 3-, and 4-day lags, and at lag 0–1 day and lag 0–4 day average concentrations prior to the death events. In general, the largest pollutant effects were observed at the lag 0–1, where pollution concentrations were evaluated at the average of the day of death (lag 0) and 1 day before death (lag 1). Therefore, for purposes of this study we focused on the results of the lag 0–1 model. All model analyses were performed using R, version 2.5.0, using the mgcv package, 1.3–24 (The R Foundation for Statistical Computing 2007).

There were two steps in the model building and fit: development of the best base model (without a pollutant) and development of the main model (with a pollutant). The latter was achieved by adding the air pollution variable(s) to the final and best cause-specific base model, assuming a linear relationship between the logarithmic mortality count and the air pollutant concentration. To obtain the best base model, the GAM analyses were performed covering two major areas. First, we controlled for potential confounding of yearly, seasonal, and subseasonal variations and for other time-varying influences on mortality. To begin, we included indicators for days of the week to take into account the change in traffic volume between workdays and weekends. We then regressed the natural logarithm of the daily death counts on a day sequence to adjust for time trends using either natural splines (ns) or penalized splines (ps). Furthermore, visual inspection of the mortality time-series showed two peaks of death counts over the two periods 28 July–3 August 2003 (sum03) and 1 December–31 December 2003 (win03). We added a factor variable for the three periods (sum03, win03, and others) and performed local smoothing by specifying the “by” option for these three periods to control for the extreme peaks of death counts. Second, we controlled for potential confounding of relevant weather variables, which is important during unusually high and low temperatures in Wuhan. We controlled for weather variables using *a*) indicator variables for extremely hot days, cold days, and humid days; and *b*) ns or ps for the temperature and humidity, respectively. The extremely hot and cold days were defined as those days on which the highest or lowest daily average temperatures were > 95th percentile or < 5th percentile of the 4 years of data, respectively (Dockery et al. 1992). The 5th and 95th percentiles for temperature were 3.6 and 31.7°C.. Similarly, the extremely humid days were those days with daily average relative humidity > 95th percentile of the 4 years of data. The goal in the previous two steps was to obtain conservative estimates on the subsequent pollution mortality associations.

Taking into account the literature review and the common protocol of the Health Effects Institute’s program of the Public Health and Air Pollution in Asia, we used four competing approaches to determine the appropriate degrees of freedom (df) for the time and weather in developing the best base model for each cause-specific mortality model (Curriero et al. 2002; Dominici et al. 2003). These include two ns methods that used the fixed df, the sequential ns method, and the ps method, where the former three ns methods were parametric-based regression splines and always used 2 df and 3 df for the local smoothers for sum03 and win03, respectively. For the two fixed-df models, we considered 6 and 8 df/year for time, 3 and 4 df for temperature, and 3 and 4 df for humidity over the entire 4-year study period. For the sequential method, we started with a reduced model (only days of week, extreme weather indicators, and local smoothing terms). We tried 3–8 df/year for the time and then chose the df that had the smallest sum of the absolute partial autocorrelation values over a 30-day lag period. Next we added temperature to the above model using 2–4 df. We repeated this process for relative humidity after including temperature, time trend, days of week, and extreme weather indicators. We ran the ps model to select the optimal df for overall time trend, local time intervals, temperature, and relative humidity. We initialized the df as 8 df/year for time, 3 df for sum03, 3 df for win03, and 3 df for both temperature and relative humidity. We observed that the local smoothing df remains the same or within 1–2 df differences from the dfs used in the sequential method for various cause-specific mortality. The criteria for selecting the best-fitting model are as follows: *a*) the absolute value of the partial autocorrelation < 0.1 for all 30-day lags; and *b*) the smallest sum of the absolute partial autocorrelation values over a 30-day lag period.

To address whether estimated effects are valid and whether they are strongly influenced by different model specifications during the modeling process, we conducted a series of sensitivity analyses in two areas. The first area concerns different smoothing approaches for time, temperature, and humidity. These included *a*) alternating smoothing order in the sequential method from time, temperature, and humidity to temperature, humidity, and time; *b*) using fixed df for time, temperature, and humidity (e.g., 6 df for time/year, 3 df for temperature, and 3 df for humidity; and 8 df for time/year, 4 df for temperature, and 4 df for humidity); and *c*) using the ps approach. The second area concerns model specifications, where the best main models were fitted alternatively by *a*) adding influenza epidemics; *b*) adding an indicator for the period of ICD-10 use; *c*) removing Wuhan, the most industrialized district; *d*) removing extreme temperature data; *e*) redefining extreme temperature; and *f*) adding the lag climate variable

Last, we redefined the temperature groups using different percentile cutoffs of the temperature ranges (3rd, 7th, 10th, and 15th percentiles) to determine whether the effects observed using the 5th percentiles were significantly changed.

We used several approaches to investigate the validity of the linearity assumption for each air pollutant. First, we replaced the linear term of the pollutant concentrations with a smooth function with 3 df using ns. Both the likelihood ratio test with 2 df (which compares the original main model with the smoothed model) and the visual inspection approach were used to assess whether the smoothed exposure–response curve resembles a straight line. Next, we performed piecewise regression models by allowing different slopes of pollutant concentrations before and after a cutoff point. The cutoff points of PM_10_ were tested from zero to 150 μg/m^3^ in 25-μg/m^3^ increments. The best piecewise regression model was the one in which the cutoff point minimized the generalized cross-validation value. In general, assuming the linearity of air pollution effects on the logarithm of mortality appears to be appropriate.

To investigate the synergetic effects between air pollution and temperature, our main models were built to include additional season indicators and two interaction terms between a linear term of air pollution and an indicator of either extreme high temperature or extreme low temperature (the normal temperature serves as the reference). The effect estimates were expressed using a percentage change in the mean number of daily deaths per 10-μg/m^3^ increments in 24-hr mean concentrations of a pollutant (8-hr mean concentrations for O_3_). The associated upper and lower 95% confidence limits by weather condition were obtained by taking the exponential of the upper and lower 95% confidence limits of the estimated βs. The overall test of the interaction effects between extreme high and low temperatures and air pollution was performed using the likelihood ratio test with 2 df.

## Results

The daily mean concentrations of PM_10_, SO_2_, and NO_2_ were much lower during high-temperature days than during low-temperature and normal-temperature days (Table 1). The 8-hr mean concentrations of O_3_, as expected, were highest during the high-temperature days. There was great variation in the daily average temperature (33.1°C vs. 2.2°C) but small variation in the daily average relative humidity among the three temperature groups.

There were considerable variations in mean daily levels of pollutants (Table 2). The mean daily concentrations of SO_2_ and NO_2_ generally increased during the study period across the three temperature groups. Despite spatial variations in the daily mean concentrations, which were mainly driven by the highest PM_10_ and SO_2_ concentrations measured at the Wugan station located near a smelter, we found that the distributions of PM_10_ over distances were fairly homogeneous, as shown by the high Pearson correlation coefficients between measurements from the monitoring stations (0.50–0.97). SO_2_ and NO_2_ were similarly homogeneously distributed except during the high-temperature days.

We collected information on a total of 89,131 nonaccidental death cases. The daily mean number of nonaccidental deaths was 61, with a maximum of 213 and with a main contribution of cardiopulmonary mortality (daily mean of 35). The majority of individuals died when they were ≥ 65 years of age (71.9%). The mean age of nonaccidental deaths was 69 years, with a range of 0–106 years. Persons ≥ 65 years of age contributed to more than half of the daily deaths for each of the underlying causes of death. The percentage of deaths in the 0–4 year age group was 1.5%. There were only 11 no-death days, all with normal temperature (Table 3). Each variance was greater than the mean, indicating that the mortality data followed the overdispersed Poisson distributions across the three temperature groups, which warrant additional control for weather and temporal trends in the data.

**Figure 1:**
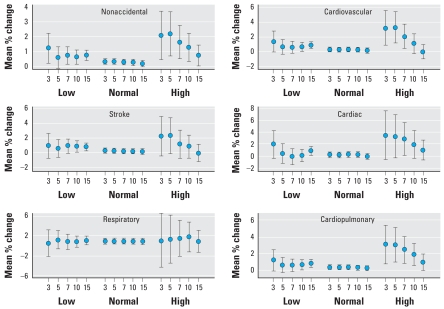
Cause-specific mortality plots for PM_10_ stratified by varying percentiles of temperature cutoff points (3, 5, 7, 10, 15) at lag 0–1 day. Values shown are the mean percentage of change in daily mortality per 10-μg/m^3^ increase in PM_10_ concentration and 95% CI.

We observed consistent associations between daily mortality and PM_10_, NO_2_, and SO_2_ (Qian et al. 2007a, 2007b). In general, using different smoothing approaches did not change the effect estimates significantly, nor did using different model specifications. We also observed a consistent interaction of PM_10_ with temperature (Table 4). The PM_10_ effects were strongest on extremely high-temperature days (daily average temperature, 33.1°C), less strong on extremely low-temperature days (2.2°C), and weakest on normal-temperature days (18.0°C). The estimates of the mean percentage of change in daily mortality per 10-μg/m^3^ increase in PM_10_ concentrations at the average of lags 0 and 1 day during high temperature were 2.20% [95% confidence interval (CI), 0.74–3.68] for nonaccidental; 3.28% (1.24–5.37) for cardiovascular; 2.35% (−0.03 to 4.78) for stroke; 3.31% (−0.22 to 6.97) for cardiac; 1.15% (−3.54 to 6.07) for respiratory; and 3.02% (1.03–5.04) for cardio-pulmonary mortality. Interestingly, we did not observe consistent stronger temperature effects of modification for the majority of outcomes in the elderly (Table 5). One possible explanation might be that the elderly were more likely to stay inside the house on hot days, avoiding exposure to extreme temperature. For the gaseous pollutants, the only interaction observed was that of O_3_ on nonaccidental mortality. We found that the estimated PM_10_ effects using the 5th percentile cutoff were generally similar to the effects estimated using the 3rd percentile (Figure 1). Except for respiratory mortality, we observed that the estimated PM_10_ effect decreased with increasing percentile on the high-temperature days. Figure 1 also shows that the relationship of daily mortality with temperature is U-shaped, which is consistent with other studies (Gouveia et al. 2003).

The estimated PM_10_ effects were attenuated in the two pollutant models (Table 6). For example, inclusion of NO_2_ in the model substantially reduced the PM_10_ effect for non-accidental mortality at normal temperature, whereas the inclusion of SO_2_ had less influence. These relationships were also present at low temperatures. Conversely, at high temperatures, the inclusion of either NO_2_ or SO_2_ had little influence on the association of PM_10_ with nonaccidental mortality. Although PM_10_ was correlated with both NO_2_ and SO_2_ (Table 7), the attenuation of the estimated effects in two-pollutant models might not be due simply to confounding, but rather an indicator of the source-related component of PM responsible for the adverse health effect. The sources and composition of PM_10_, and hence the toxicity, vary with temperature. Thus, temperature may be serving as an indicator of PM_10_ composition. The interaction of O_3_ on nonaccidental mortality was attenuated but remained significant after controlling for PM_10_ and SO_2_ in the copollutant models (Table 8). Because temperature was positively correlated with O_3_ (*r* = 0.52), part of the interaction between PM_10_ and high temperature might be due to O_3_.

## Discussion and Conclusion

We observed that high temperatures enhanced PM_10_ mortality effects, even though PM_10_ daily concentrations were lower on the extremely high-temperature days than on the normal-temperature and low-temperature days.

The small number of previous relevant studies reported conflicting results on this interaction. Samet et al. (1998) found no significant evidence that weather variables modified the pollution–mortality relationship. However, Katsouyanni et al. (1993) found a significant effect of the interaction between SO_2_ and high temperature on total mortality but no significant interactions between high temperature and either smoke or O_3_. We speculate that the following environmental features are related to the significant synergistic effects of PM_10_ and high temperature in Wuhan. First, the maximum summer temperature often exceeded 40°C and lasted about 2 weeks. Wuhan’s special topography causes narrow differences in daily high and low temperatures. Even around midnight in the summer, indoor air temperatures > 32°C are not uncommon. Thus, the city residents were exposed to high temperatures for longer periods than residents of many other cities. Second, few residences in Wuhan were built with energy conservation in mind; a vast amount of radiant energy can easily infiltrate buildings and be absorbed, even when all windows are closed. The temperature inside is commonly comparable to the temperature in the shade outside. In addition, air conditioners have seldom been used because of the high cost of electricity. Third, the most commonly used means for cooling are fans, which can be effective in protecting against heat stress in areas without extremely high temperatures. However, with the temperatures in Wuhan, the use of fans could contribute to heat stress by exacerbating dehydration (Centers for Disease Control and Prevention 1995). Finally, approximately 4.5 million permanent residents plus approximately 1 million transients live in the urban core districts with an area of 201 km^2^. This high population density adds to the urban “heat island” effect, which would make the temperature somewhat higher in the urban core areas than in the suburban areas.

The mechanism underlying the synergistic effects of ambient particle pollution and extremely high temperatures on daily mortality is not yet clear. Some potential explanations have been proposed, especially for the elderly (Easterling et al. 2000). Brunekreef and Holgate (2002) hypothesized that air particles increase the risk of cardiopulmonary mortality through direct and indirect pathophysiologic mechanisms, including pulmonary and systemic inflammation, accelerated atherosclerosis, altered cardiac autonomic function, and increase of inflammatory cytokines in the heart. Many studies have addressed the mechanisms by which high temperature is associated with increased mortality. In animal studies, Keatinge et al. (1986) observed dehydration, increased intracranial and arterial hypertension, endothelial cell damage, and cerebral ischemia during the onset of heat stroke in animals exposed to high temperatures. In a clinical trial study, Gordon et al. (1988) found that exposure to high temperatures increased plasma viscosity and serum cholesterol level. Tsai et al. (2003) suggested that high temperature may help precipitate coronary artery disease and cerebral infarction. Flynn et al. (2005) observed that many of the elderly who died in the heat wave in France during the first 2 weeks of August 2003 were dehydrated, hypernatremic, and hyperkalemic, with evidence of renal failure (Vanhems et al. 2003). The investigators postulated that the most probable causes of death during the heat wave were thromboembolic disease and malignant cardiac arrhythmias as well as heat-induced sepsislike shock (Flynn et al. 2005).

Our study has several limitations. First, both ICD-9 and ICD-10 codes were used. The change in ICD coding might produce misclassification in cause-specific mortality. To address this uncertainty, we examined daily death counts between ICD-9 and ICD-10 mortality data in 2002. We found high concordance rates between the two-coded mortality data, and the maximum change in the estimated pollution mortality effect was 0.09%. These results support our contention that the change in the ICD coding system did not significantly affect the associations identified in this study. Second, there might be other important unknown and unmeasured factors. For example, socioeconomic status can play an important role as an effect modifier. Unfortunately, we do not currently have data on hand to explore the effects of these factors. Third, interpretation of the effects of interaction between O_3_ and temperature requires caution, because O_3_ data were obtained from only one monitoring station. The limited O_3_ data may also restrict our ability to reach any reliable conclusion. Last, measurement errors in exposure are clearly applicable to this study. However, this measurement error generally belongs to the Berkson type and thus is nondifferential in nature, which is likely to cause a bias toward the null and lead to underestimated associations (Armstrong 1998).

In conclusion, we found synergistic effects between PM_10_ and extremely high temperature on daily mortality in this highly polluted city. Further studies are needed to confirm these findings.

## Figures and Tables

**Table 1:** Distributions of mean daily ambient air pollutants (μg/m^3^) and weather variables by temperature^a^ in Wuhan, China, July 2001–June 2004.

	Normal temperature		Low temperature		High temperature	
Pollutant	Days (*n*)	Mean ± SD	Days (*n*)	Mean ± SD	Days (*n*)	Mean ± SD
PM_10_	1,312	145.7 ± 64.6	73	117.3 ± 49.5	73	96.3 ± 27.9
O_3_	1,265	87.4 ± 47.5	72	51.5 ± 24.5	49	91.9 ± 41.8
SO_2_	1,311	39.4 ± 25.4	73	50.3 ± 26.7	73	23.8 ± 10.2
NO_2_	1,311	52.9 ± 18.7	73	51.2 ± 17.8	73	32.5 ± 6.2
Daily mean temperature (°C)	1,315	18.0 ± 8.2	73	2.2 ± 1.3	73	33.1 ± 0.9
Daily mean relative humidity (%)	1,315	74.4 ± 12.4	73	75.3 ± 16.0	73	64.7 ± 5.6

a Normal temperature ≥ 5th percentile and ≤ 95th percentile of daily average temperatures during the 4-year study period; low temperature < 5th percentile; high temperature > 95th percentile.

**Table 2:** Correlations and trends in measured ambient air pollutants by temperature in Wuhan, China, July 2001–June 2004.

					Mean of daily means	
Pollutant (μg/m^3^)	No. of monitoring stations	Range of mean values between stations	Coefficient of variation of daily mean (%)	Range of Pearson correlation coefficients between monitoring stations	Mean	Average annual change^a^
PM_10_						
Normal temperature	5	116.9–166.1	44.3	0.83–0.97	145.7	−4.5
Low temperature	5	95.5–126.6	42.2	0.76–0.97	117.3	4.3
High temperature	5	72.7–118.6	28.9	0.50–0.93	96.3	−1.5
O_3_						
Normal temperature	1	NA	54.3	NA	87.4	−2.8
Low temperature	1	NA	47.7	NA	51.5	4.6
High temperature	1	NA	45.5	NA	91.9	−3.0
SO_2_						
Normal temperature	4	32.8–45.9	64.4	0.64–0.84	39.4	3.3
Low temperature	4	41.3–58.7	53.0	0.61–0.87	50.3	4.0
High temperature	4	17.4–28.1	42.9	0.27–0.78	23.8	2.6
NO_2_						
Normal temperature	5	36.3–64.8	35.3	0.57–0.84	52.9	2.1
Low temperature	5	37.6–61.9	34.8	0.69–0.86	51.2	3.3
High temperature	5	22.3–43.2	19.1	0.11–0.66	32.5	1.3

NA, not applicable.

a Calculated from a linear regression model.

**Table 3:** Daily mortality in Wuhan, China, by cause of death and temperature, July 2001–June 2004.

						Percentile				
Underlying cause of death	Total no.of deaths	No. of days with no deaths	Mean	Variance	Variance/mean	Minimum	Maximum	25th	50th	75th
Nonaccidental										
Normal temperature	78,666	0	59.82	216.23	3.61	25	213	50	58	67
Low temperature	5,839	0	79.99	142.96	1.79	57	107	71	80	88
High temperature	4,626	0	63.37	562.10	8.87	40	156	51	56	68
Cardiovascular										
Normal temperature	35,684	0	27.14	65.75	2.42	8	67	21	26	32
Low temperature	2,815	0	38.56	56.78	1.47	26	60	33	37	43
High temperature	2,124	0	29.10	194.73	6.69	11	94	22	26	32
Stroke										
Normal temperature	22,544	0	17.14	31.24	1.82	4	43	13	17	21
Low temperature	1,713	0	23.47	25.97	1.11	14	35	20	23	27
High temperature	1,300	0	17.81	71.27	4.00	6	57	13	16	20
Cardiac										
Normal temperature	10,634	2	8.09	12.09	1.50	0	22	6	8	10
Low temperature	898	0	12.30	16.88	1.37	3	23	9	12	15
High temperature	634	0	8.68	25.11	2.89	2	29	5	8	11
Respiratory										
Normal temperature	8,894	9	6.76	32.14	4.75	0	125	4	6	8
Low temperature	894	0	12.25	15.86	1.29	5	25	9	13	15
High temperature	499	0	6.84	46.50	6.80	1	56	4	5	8
Cardiopulmonary										
Normal temperature	44,578	0	33.90	137.88	4.07	11	185	26	32	39
Low temperature	3,709	0	50.81	87.88	1.73	33	78	44	50	56
High temperature	2,623	0	35.93	345.09	9.60	15	111	27	32	38

**Table 4:** Estimates of the mean percentage of change (95% CI) in daily mortality per 10-μg/m^3^ increase in pollutants by cause of death and temperature, lag 0–1 day, in Wuhan, China, July 2001–June 2004.

	Temperature			
Cause of death	Normal	Low	High	*p*-Value
Nonaccidental				
PM_10_	0.36 (0.17 to 0.56)	0.62 (−0.09 to 1.34)	2.20 (0.74 to 3.68)	0.014
NO_2_	1.89 (1.22 to 2.57)	2.22 (0.16 to 4.32)	4.59 (−1.78 to 11.36)	0.613
SO_2_	1.10 (0.55 to 1.66)	1.74 (0.25 to 3.26)	2.56 (−2.11 to 7.45)	0.505
O_3_	0.19 (−0.15 to 0.54)	0.68 (−0.83 to 2.21)	1.41 (0.23 to 2.61)	0.049
Cardiovascular				
PM_10_	0.39 (0.11 to 0.66)	0.72 (−0.25 to 1.70)	3.28 (1.24 to 5.37)	0.007
NO_2_	1.89 (0.95 to 2.84)	2.03 (−0.78 to 4.92)	5.23 (−3.71 to 15.00)	0.727
SO_2_	1.36 (0.57 to 2.15)	1.81 (−0.24 to 3.91)	0.35 (−6.18 to 7.32)	0.840
O_3_	−0.25 (−0.72 to 0.22)	0.09 (−1.94 to 2.15)	1.39 (−0.25 to 3.06)	0.092
Stroke				
PM_10_	0.38 (0.06 to 0.70)	0.67 (−0.50 to 1.85)	2.35 (−0.03 to 4.78)	0.222
NO_2_	1.94 (0.82 to 3.06)	2.02 (−1.35 to 5.50)	4.42 (−5.96 to 15.95)	0.895
SO_2_	0.99 (0.06 to 1.92)	1.32 (−1.12 to 3.82)	−0.26 (−8.01 to 8.14)	0.913
O_3_	−0.27 (−0.81 to 0.28)	0.57 (−1.91 to 3.10)	1.09 (−0.77 to 2.98)	0.275
Cardiac				
PM_10_	0.32 (−0.14 to 0.79)	0.50 (−1.10 to 2.13)	3.31 (−0.22 to 6.97)	0.229
NO_2_	1.92 (0.31 to 3.55)	1.17 (−3.44 to 6.00)	−0.31 (−14.58 to 16.35)	0.911
SO_2_	2.04 (0.70 to 3.39)	1.90 (−1.50 to 5.41)	−1.99 (−12.65 to 9.98)	0.771
O_3_	−0.64 (−1.44 to 0.16)	−0.04 (−3.39 to 3.42)	1.45 (−1.47 to 4.46)	0.332
Respiratory				
PM_10_	0.80 (0.25 to 1.35)	1.07 (−0.76 to 2.95)	1.15 (−3.54 to 6.07)	0.931
NO_2_	3.64 (1.69 to 5.63)	3.17 (−2.13 to 8.75)	7.68 (−12.36 to 32.30)	0.896
SO_2_	1.84 (0.29 to 3.41)	2.84 (−0.99 to 6.82)	12.75 (−2.59 to 30.51)	0.253
O_3_	−0.06 (−1.09 to 0.99)	1.14 (−2.88 to 5.33)	2.98 (−0.79 to 6.90)	0.160
Cardiopulmonary				
PM_10_	0.45 (0.19 to 0.70)	0.69 (−0.22 to 1.61)	3.02 (1.03 to 5.04)	0.014
NO_2_	2.13 (1.24 to 3.03)	1.98 (−0.65 to 4.68)	4.31 (−4.32 to 13.72)	0.852
SO_2_	1.28 (0.56 to 2.01)	1.43 (−0.46 to 3.36)	2.26 (−4.05 to 8.98)	0.930
O_3_	0.04 (−0.42 to 0.50)	−0.01 (−1.89 to 1.92)	1.51 (−0.11 to 3.16)	0.123

**Table 5:** Estimates of the mean percentage of change (95% CI) in daily mortality per 10-μg/m^3^ increase in PM_10_ concentration by cause of death, temperature, and age, lag 0–1 day, in Wuhan, China, July 2001–June 2004.

	Temperature			
Cause of death, age (years)	Normal	Low	High	*p*-Value
Nonaccidental				
< 65	0.23 (−0.10 to 0.56)	1.78 (0.52 to 3.05)	2.34 (−0.09 to 4.83)	0.010
≥ 65	0.41 (0.18 to 0.64)	0.22 (−0.61 to 1.05)	2.14 (0.42 to 3.89)	0.071
Cardiovascular				
< 65	0.17 (−0.40 to 0.73)	2.63 (0.67 to 4.63)	4.32 (0.10 to 8.71)	0.007
≥ 65	0.44 (0.14 to 0.74)	0.24 (−0.84 to 1.32)	3.03 (0.77 to 5.34)	0.043
Stroke				
< 65	0.17 (−0.53 to 0.88)	2.85 (0.34 to 5.42)	4.54 (−0.79 to 10.16)	0.031
≥ 65	0.43 (0.07 to 0.79)	0.11 (−1.22 to 1.45)	1.83 (−0.83 to 4.57)	0.489
Cardiac				
< 65	−0.04 (−1.07 to 1.01)	1.79 (−1.65 to 5.35)	2.71 (−4.58 to 10.56)	0.458
≥ 65	0.40 (−0.10 to 0.91)	0.19 (−1.55 to 1.95)	3.45 (−0.41 to 7.46)	0.292
Respiratory				
< 65	−0.35 (−1.85 to 1.18)	−1.13 (−6.33 to 4.35)	−3.42 (−15.82 to 10.80)	0.856
≥ 65	0.93 (0.38 to 1.50)	1.30 (−0.57 to 3.20)	1.76 (−3.03 to 6.78)	0.852
Cardiopulmonary				
< 65	0.07 (−0.47 to 0.61)	1.95 (0.04 to 3.90)	3.49 (−0.66 to 7.81)	0.040
≥ 65	0.53 (0.25 to 0.81)	0.43 (−0.57 to 1.44)	2.91 (0.74 to 5.12)	0.052

**Table 6:** Copollutant regression estimates of the mean percentage of change (95% CI) in daily mortality per 10-μg/m^3^ increase in PM_10_ concentration by temperature, lag 0–1 day, in Wuhan, China, July 2001–June 2004.

	Temperature		
Cause of death, pollutant	Normal	Low	High
Nonaccidental			
PM_10_	0.36 (0.17 to 0.56)	0.62 (−0.09 to 1.34)	2.20 (0.74 to 3.68)
PM_10_ + NO_2_	0.07 (−0.17 to 0.30)	0.24 (−0.49 to 0.97)	1.87 (0.42 to 3.35)
PM_10_ + SO_2_	0.27 (0.06 to 0.47)	0.45 (−0.27 to 1.17)	2.12 (0.67 to 3.60)
PM_10_ + O_3_	0.38 (0.18 to 0.58)	0.72 (0.00 to 1.44)	2.15 (0.55 to 3.77)
Cardiovascular			
PM_10_	0.39 (0.11 to 0.66)	0.72 (−0.25 to 1.70)	3.28 (1.24 to 5.37)
PM_10_ + NO_2_	0.11 (−0.23 to 0.45)	0.37 (−0.62 to 1.38)	3.00 (0.95 to 5.09)
PM_10_ + SO_2_	0.27 (−0.02 to 0.55)	0.50 (−0.47 to 1.49)	3.20 (1.16 to 5.29)
PM_10_ + O_3_	0.42 (0.15 to 0.70)	0.82 (−0.16 to 1.80)	3.71 (1.50 to 5.96)
Stroke			
PM_10_	0.38 (0.06 to 0.70)	0.67 (−0.50 to 1.85)	2.35 (−0.03 to 4.78)
PM_10_ + NO_2_	0.09 (−0.31 to 0.49)	0.29 (−0.90 to 1.51)	2.05 (−0.34 to 4.49)
PM_10_ + SO_2_	0.31 (−0.03 to 0.64)	0.53 (−0.65 to 1.73)	2.31 (−0.07 to 4.74)
PM_10_ + O_3_	0.38 (0.05 to 0.71)	0.69 (−0.48 to 1.87)	2.77 (0.25 to 5.35)
Cardiac			
PM_10_	0.32 (−0.14 to 0.79)	0.50 (−1.10 to 2.13)	3.31 (−0.22 to 6.97)
PM_10_ + NO_2_	0.02 (−0.57 to 0.60)	0.12 (−1.53 to 1.80)	3.01 (−0.54 to 6.69)
PM_10_ + SO_2_	0.11 (−0.38 to 0.61)	0.14 (−1.48 to 1.78)	3.17 (−0.37 to 6.84)
PM_10_ + O_3_	0.41 (−0.06 to 0.89)	0.72 (−0.90 to 2.37)	4.92 (0.96 to 9.03)
Respiratory			
PM_10_	0.80 (0.25 to 1.35)	1.07 (−0.76 to 2.95)	1.15 (−3.54 to 6.07)
PM_10_ + NO_2_	0.30 (−0.39 to 0.99)	0.44 (−1.46 to 2.36)	0.63 (−4.07 to 5.55)
PM_10_ + SO_2_	0.64 (0.07 to 1.22)	0.80 (−1.05 to 2.69)	1.03 (−3.66 to 5.94)
PM_10_ + O_3_	0.84 (0.28 to 1.41)	1.11 (−0.73 to 2.99)	2.66 (−2.44 to 8.02)
Cardiopulmonary			
PM_10_	0.45 (0.19 to 0.70)	0.69 (−0.22 to 1.61)	3.02 (1.03 to 5.04)
PM_10_ + NO_2_	0.15 (−0.17 to 0.47)	0.33 (−0.61 to 1.27)	2.70 (0.72 to 4.73)
PM_10_ + SO_2_	0.34 (0.07 to 0.61)	0.50 (−0.42 to 1.43)	2.95 (0.96 to 4.97)
PM_10_ + O_3_	0.43 (0.17 to 0.70)	0.76 (−0.16 to 1.68)	3.32 (1.16 to 5.53)

**Table 7:** Pearson correlations between daily measurements of pollutants in Wuhan, China, stratified by temperature, July 2001–June 2004.

Temperature, pollutant	NO_2_	SO_2_	O_3_
Normal			
PM_10_	0.72	0.59	0.06
NO_2_		0.75	0.04
SO_2_			0.01
Low			
PM_10_	0.83	0.74	0.19
NO_2_		0.87	0.31
SO_2_			0.33
High			
PM_10_	0.68	0.15	0.65
NO_2_		0.45	0.65
SO_2_			0.42

**Table 8:** Copollutant regression estimates of the mean percentage of change (95% CI) in daily mortality per 10*-*μg/m^3^ increase in O_3_ concentrations by temperature, lag 0–1 day mean, in Wuhan, China, July2001–June 2004.

	Temperature		
Cause of death, pollutant	Normal	Low	High
Nonaccidental			
O_3_	0.19 (−0.15 to 0.54)	0.68 (−0.83 to 2.21)	1.41 (0.23 to 2.61)
O_3_ + PM_10_	0.16 (−0.18 to 0.50)	0.52 (−0.98 to 2.04)	1.20 (0.02 to 2.39)
O_3_ + NO_2_	0.02 (−0.33 to 0.36)	0.33 (−1.16 to 1.85)	1.10 (−0.07 to 2.29)
O_3_ + SO_2_	0.06 (−0.29 to 0.41)	0.38 (−1.12 to 1.90)	1.25 (0.07 to 2.44)
Cardiovascular			
O_3_	−0.25 (−0.72 to 0.22)	0.09 (−1.94 to 2.15)	1.39 (−0.25 to 3.06)
O_3_ + PM_10_	−0.25 (−0.71 to 0.22)	0.00 (−2.01 to 2.06)	1.16 (−0.47 to 2.82)
O_3_ + NO_2_	−0.39 (−0.86 to 0.08)	−0.20 (−2.22 to 1.85)	1.09 (−0.54 to 2.74)
O_3_ + SO_2_	−0.37 (−0.84 to 0.10)	−0.21 (−2.23 to 1.85)	1.22 (−0.41 to 2.88)
Stroke			
O_3_	−0.27 (−0.81 to 0.28)	0.57 (−1.91 to 3.10)	1.09 (−0.77 to 2.98)
O_3_ + PM_10_	−0.28 (−0.82 to 0.26)	0.48 (−1.99 to 3.01)	0.87 (−0.98 to 2.76)
O_3_ + NO_2_	−0.42 (−0.97 to 0.13)	0.27 (−2.19 to 2.80)	0.78 (−1.07 to 2.66)
O_3_ + SO_2_	−0.37 (−0.92 to 0.18)	0.37 (−2.11 to 2.90)	0.96 (−0.89 to 2.85)
Cardiac			
O_3_	−0.64 (−1.44 to 0.16)	−0.04 (−3.39 to 3.42)	1.45 (−1.47 to 4.46)
O_3_ + PM_10_	−0.61 (−1.41 to 0.19)	−0.17 (−3.51 to 3.28)	1.26 (−1.66 to 4.27)
O_3_ + NO_2_	−0.77 (−1.57 to 0.04)	−0.40 (−3.74 to 3.06)	1.16 (−1.76 to 4.16)
O_3_ + SO_2_	−0.82 (−1.62 to –0.01)	−0.58 (−3.91 to 2.86)	1.20 (−1.71 to 4.19)
Respiratory			
O_3_	−0.06 (−1.09 to 0.99)	1.14 (−2.88 to 5.33)	2.98 (−0.79 to 6.90)
O_3_ + PM_10_	−0.06 (−1.09 to 0.98)	0.84 (−3.16 to 5.02)	2.57 (−1.19 to 6.48)
O_3_ + NO_2_	−0.37 (−1.41 to 0.67)	0.53 (−3.48 to 4.71)	2.41 (−1.34 to 6.31)
O_3_ + SO_2_	−0.27 (−1.31 to 0.79)	0.65 (−3.37 to 4.83)	2.72 (−1.04 to 6.63)
Cardiopulmonary			
O_3_	0.04 (−0.42 to 0.50)	−0.01 (−1.89 to 1.92)	1.51 (−0.11 to 3.16)
O_3_ + PM_10_	−0.01 (−0.46 to 0.45)	−0.22 (−2.10 to 1.69)	1.37 (−0.24 to 3.00)
O_3_ + NO_2_	−0.18 (−0.63 to 0.29)	−0.45 (−2.32 to 1.46)	1.26 (−0.34 to 2.89)
O_3_ + SO_2_	−0.13 (−0.60 to 0.34)	−0.38 (−2.26 to 1.54)	1.45 (−0.16 to 3.08)
Noncardiopulmonary			
O_3_	0.22 (−0.22 to 0.66)	1.39 (−0.74 to 3.57)	0.50 (−1.01 to 2.02)
O_3_ + PM_10_	0.21 (−0.23 to 0.65)	1.26 (−0.87 to 3.42)	0.37 (−1.13 to 1.90)
O_3_ + NO_2_	0.09 (−0.35 to 0.54)	1.10 (−1.03 to 3.26)	0.30 (−1.20 to 1.82)
O_3_ + SO_2_	0.14 (−0.31 to 0.58)	1.12 (−1.01 to 3.29)	0.41 (−1.10 to 1.94)
